# Finerenone, a Non-Steroidal Mineralocorticoid Receptor Antagonist, Reduces Vascular Injury and Increases Regulatory T-Cells: Studies in Rodents with Diabetic and Neovascular Retinopathy

**DOI:** 10.3390/ijms24032334

**Published:** 2023-01-25

**Authors:** Jack R. Jerome, Devy Deliyanti, Varaporn Suphapimol, Peter Kolkhof, Jennifer L. Wilkinson-Berka

**Affiliations:** 1 Department of Anatomy and Physiology, School of Biomedical Sciences, The University of Melbourne, Parkville, VIC 3010, Australia; 2 Bayer AG, 42113 Wuppertal, Germany

**Keywords:** finerenone, mineralocorticoid receptor, diabetic retinopathy, angiogenesis, Tregs

## Abstract

Vision loss in diabetic retinopathy features damage to the blood–retinal barrier and neovascularization, with hypertension and the renin–angiotensin system (RAS) having causal roles. We evaluated if finerenone, a non-steroidal mineralocorticoid receptor (MR) antagonist, reduced vascular pathology and inflammation in diabetic and neovascular retinopathy. Diabetic and hypertensive transgenic (mRen-2)27 rats overexpressing the RAS received the MR antagonist finerenone (10 mg/kg/day, oral gavage) or the angiotensin-converting enzyme inhibitor perindopril (10 mg/kg/day, drinking water) for 12 weeks. As retinal neovascularization does not develop in diabetic rodents, finerenone (5 mg/kg/day, i.p.) was evaluated in murine oxygen-induced retinopathy (OIR). Retinal vasculopathy was assessed by measuring gliosis, vascular leakage, neovascularization, and VEGF. Inflammation was investigated by quantitating retinal microglia/macrophages, pro-inflammatory mediators, and anti-inflammatory regulatory T-cells (Tregs). In diabetes, both treatments reduced systolic blood pressure, gliosis, vascular leakage, and microglial/macrophage density, but only finerenone lowered VEGF, ICAM-1, and IL-1ß. In OIR, finerenone reduced neovascularization, vascular leakage, and microglial density, and increased Tregs in the blood, spleen, and retina. Our findings, in the context of the FIDELIO-DKD and FIGARO-DKD trials reporting the benefits of finerenone on renal and cardiovascular outcomes in diabetic kidney disease, indicate the potential of finerenone as an effective oral treatment for diabetic retinopathy.

## 1. Introduction

The mineralocorticoid (MR) has a significant role in maintaining blood pressure and electrolyte homeostasis via its principal ligand aldosterone [1,2]. However, pre-clinical and clinical studies have identified that overactivation of aldosterone/MR and the renin–angiotensin system (RAS) is involved in the pathogenesis of various diseases including hypertension [3], coronary artery disease [4], chronic kidney disease [5], and congestive heart failure [6]. Aldosterone/MR contributes to these pathologies by stimulating fibrotic, inflammatory, and oxidative stress pathways, amongst others [7,8,9,10]. Hence, antagonism of the MR has been extensively studied as a treatment approach.

The MR is an intracellular steroid hormone receptor and a member of the nuclear receptor superfamily of proteins. The development of the steroidal MR antagonists spironolactone and eplerenone led to convincing evidence from pre-clinical studies that MR blockade reduces tissue damage in various diseases [11,12,13,14]. However, their use in patients, and particularly those with chronic kidney disease, has raised concerns due to the high incidence of hyperkalemia [15]. Finerenone is a novel non-steroidal MR antagonist, which allows it to bind with high affinity to the MR and inhibit the recruitment of transcriptional co-activators involved in the expression of pathogenic genes more effectively than steroidal MR antagonists [16,17]. These properties of finerenone and the absence of unwanted side-effects due to other hormonal stimulation of the MR has led to substantial interest in its therapeutic potential [18]. The recent clinical trials FIDELIO-DKD (Finerenone in Reducing Kidney Failure and Disease Progression in Diabetic Kidney Disease) and FIGARO-DKD (Finerenone in Reducing Cardiovascular Mortality and Morbidity in Diabetic Kidney Disease) included more than 13,000 patients with chronic kidney disease and type-2 diabetes [19,20]. The results of FIDELIO-DKD and FIGARO-DKD provide an important advance in the field, with finerenone significantly reducing kidney and cardiovascular outcomes [19,20], indicating that finerenone might have therapeutic benefit in other diabetic complications such as diabetic retinopathy (DR) [21].

DR is a leading cause of vision loss and blindness across the globe that is escalating in prevalence [22]. Progressive damage to the retinal vasculature resulting in breakdown of the blood–retinal barrier (BRB) is a clinical hallmark of DR. It involves the excessive up-regulation of mediators such as vascular endothelial growth factor (VEGF) which promote vascular permeability, oedema, and neovascularization [23]. Inflammation is a key contributor to retinal vascular pathology involving pro-inflammatory retinal microglia of the innate immune system [24,25], and there is emerging evidence of a role for T-lymphocytes of the adaptive immune system [26,27]. There is evidence from pre-clinical studies that aldosterone/MR contributes to retinal vasculopathy and inflammation, with spironolactone and eplerenone reducing breakdown of the BRB, neovascularization, and inflammation [28,29]. However, the ability of finerenone to attenuate DR has not been investigated.

The aim of the present study was to determine if finerenone reduced retinal vascular disease and inflammation in animal models of diabetic and neovascular retinopathy. Hypertension is a risk factor for DR and associated with overactivity of the RAS [30]. Therefore, our studies were performed in diabetic and transgenic (mRen-2)27 rats that are hypertensive due to overexpression of renin in extrarenal tissues and develop cardiorenal disease and hypertensive retinopathy [31,32,33,34,35]. As diabetic rodents do not progress to proliferative DR, we evaluated the oxygen-induced retinopathy (OIR) model, which develops extensive retinal neovascularization. We previously reported that retinal vasculopathy and inflammation in OIR is reduced by increasing the trafficking of anti-inflammatory Forkhead protein box P3 regulatory T-cells (Foxp3^+^ Tregs) into the retina [26]. Here, we investigated if finerenone influenced the abundance of Foxp3^+^ Tregs in mice with OIR.

## 2. Results

### 2.1. Diabetes: Body Weight, Blood Glucose, and Systolic Blood Pressure

**Figure 1:**
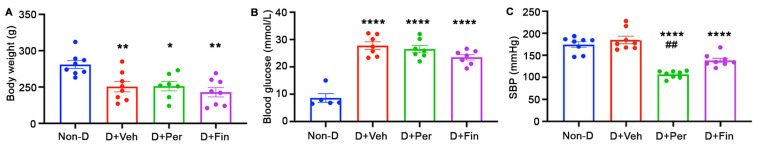
Body weight, blood glucose levels, and systolic blood pressure in (mRen-2)27 rats after 12 weeks of diabetes. D, diabetic. Veh, vehicle. Per, perindopril. Fin, finerenone. (**A**) Body weight. (**B**) Blood glucose levels. (**C**) Systolic blood pressure (SBP). * *p* < 0.05, ** *p* < 0.01, **** *p* < 0.0001 to non-diabetic. ## *p* < 0.01 to diabetic+finerenone. *n* = 5 to 8 rats per group. Values are mean ± SEM.

Diabetes reduced body weight and increased blood glucose levels in (mRen-2)27 rats but had no effect on systolic blood pressure compared to non-diabetic controls (Figure 1). In diabetic (mRen-2)27 rats, oral treatment with finerenone was compared to the angiotensin-converting enzyme inhibitor perindopril, delivered in drinking water for 12 weeks. Finerenone and perindopril did not influence body weight and blood glucose levels but reduced systolic blood pressure compared to non-diabetic controls (Figure 1). Perindopril reduced systolic blood pressure to a greater extent than finerenone.

### 2.2. Diabetes: Finerenone Reduced Damage to the Retinal Vasculature

**Figure 2:**
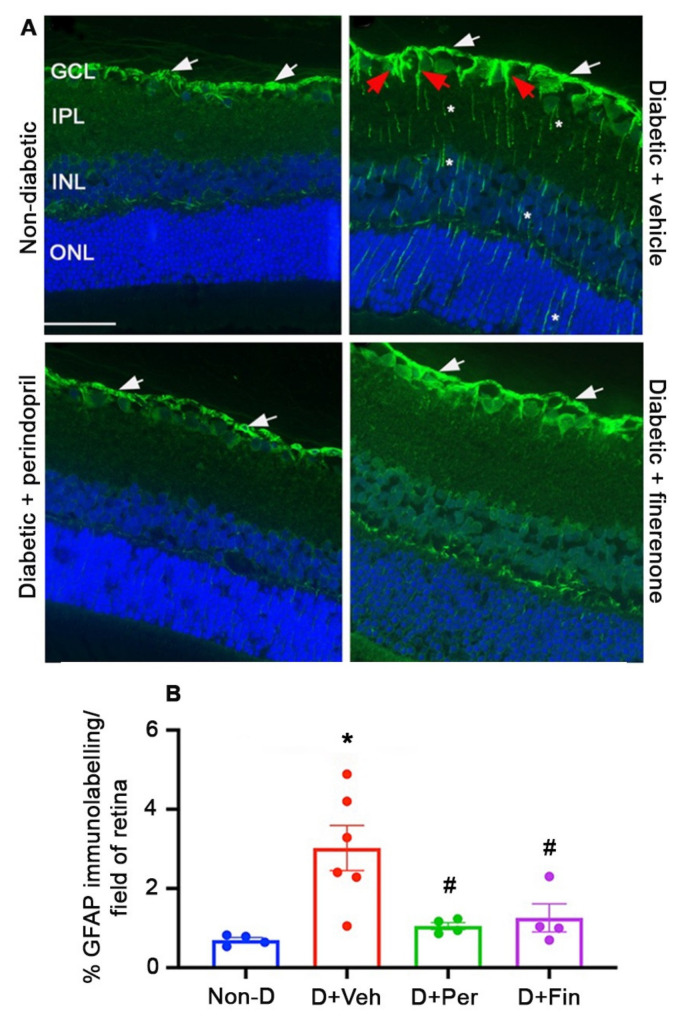
Finerenone reduced GFAP immunolabeling in (mRen-2)27 rats after 12 weeks of diabetes. Three-micrometer sections of retina immunolabeled with GFAP (green) and counterstained with DAPI to identify nuclei (blue). Scale bar = 50 µm. D, diabetic. Veh, vehicle. Per, perindopril. Fin, finerenone. GCL, ganglion cell layer. IPL, inner plexiform layer. INL, inner nuclear layer. ONL, outer nuclear layer. (**A**) GFAP immunolabeling at the retinal surface (white arrows) and the endfeet (red arrows) and cell processes (asterisks) of macroglial Müller cells. (**B**) GFAP quantitation. * *p* < 0.05 to non-diabetic. # *p* < 0.05 to diabetic+vehicle. *n* = 4 to 6 rats per group. Values are mean ± SEM.

Macroglial Müller cells and astrocytes within the retina assist in the maintenance of the BRB and when damaged obtain a gliotic phenotype [36]. Gliosis was evaluated with immunohistochemistry for glial fibrillary acidic protein (GFAP) using an established method [37]. In (mRen-2)27 rats, diabetes increased GFAP immunolabeling at the surface of the retina and in Müller cells compared to non-diabetic controls (Figure 2). Treatment with perindopril and finerenone reduced GFAP immunolabeling to the level of non-diabetic rats (Figure 2).

### 2.3. Diabetes: Finerenone Reduced VEGF and Retinal Vascular Leakage

**Figure 3:**
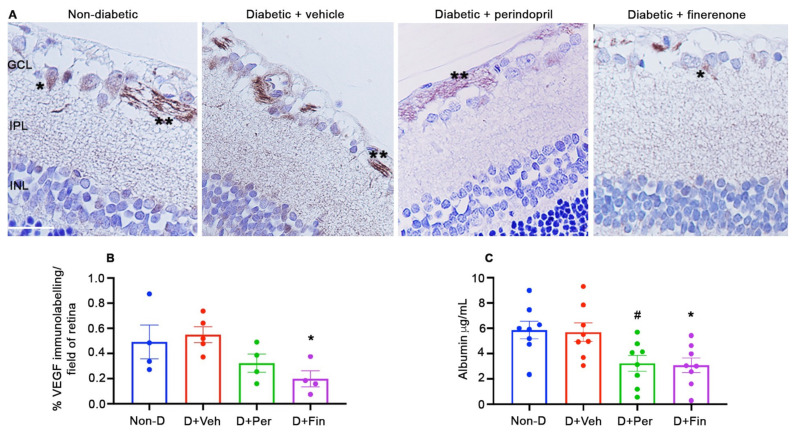
Finerenone reduced VEGF and vascular leakage in (mRen-2)27 after 12 weeks of diabetes. D, diabetic. Veh, vehicle. Per, perindopril. Fin, finerenone. (**A**) Three-micrometer paraffin sections of retina immunolabeled with VEGF and counterstained with hematoxylin. GCL, ganglion cell layer. IPL, inner plexiform layer. INL, inner nuclear layer. Scale bar = 40 µm. Ganglion cells denoted by single asterisk (*). Glial cells denoted by double asterisks (**). (**B**) Quantitation of VEGF immunolabeling in the retina. * *p* < 0.05 to diabetic+vehicle. *n* = 4 to 5 rats per group. (**C**) Vitreal levels of albumin (ELISA). * *p* < 0.05 to non-diabetic and diabetic + vehicle. # *p* < 0.05 to non-diabetic. *n* = 7 to 8 rats per group. Values are mean ± SEM.

In response to diabetes and tissue stress, Müller cells and neuronal ganglion cells increase their expression of VEGF [38,39]. In non-diabetic (mRen-2)27 rats, VEGF immunolabeling was present in the endfeet of Müller cells and the cytoplasm of ganglion cells. The extent of VEGF immunolabeling was similar between non-diabetic controls and diabetic controls (Figure 3). In diabetic rats, finerenone but not perindopril reduced VEGF immunolabeling compared to non-diabetic and diabetic controls (Figure 3). We next evaluated if finerenone influenced the permeability of the BRB by measuring the levels of albumin sequestered into the vitreous cavity. As for VEGF immunolabeling, vascular leakage was similar between non-diabetic and diabetic controls (Figure 3). In diabetic rats, finerenone reduced vitreal albumin levels compared to non-diabetic and diabetic rats, while perindopril reduced vitreal albumin levels compared to non-diabetic rats (Figure 3).

### 2.4. Diabetes: Finerenone Reduced Retinal Inflammation

**Figure 4:**
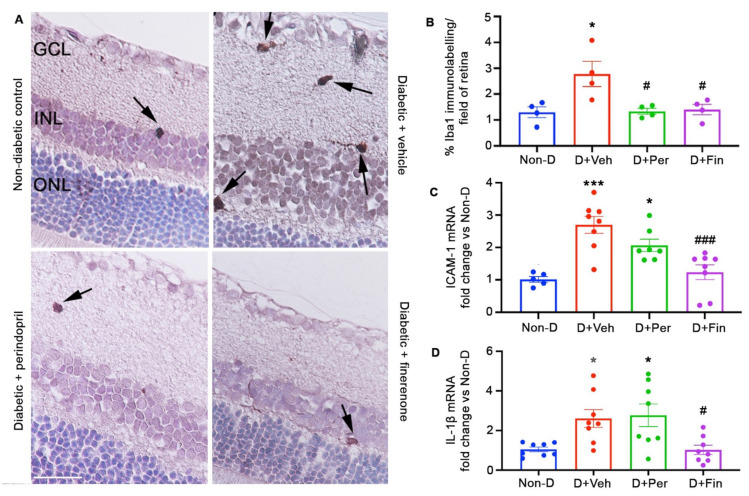
Finerenone reduced microglia/macrophage density and pro-inflammatory mediators in the retina of (mRen-2)27 rats after 12 weeks of diabetes. D, diabetic. Veh, vehicle. Per, perindopril. Fin, finerenone. (**A**) Three-micrometer paraffin sections of retina immunolabeled with Iba1 to detect microglia/macrophages. Counterstain, hematoxylin. Scale bar = 50 µm. Iba1 labelled microglia/macrophages are denoted by arrows. (**B**) Iba1 quantitation. * *p* < 0.05 to non-diabetic. # *p* < 0.05 to diabetic+vehicle. *n* = 4 rats per group. (**C**) Retinal ICAM-1 mRNA levels. (**D**) Retinal IL-1ß mRNA levels. * *p* < 0.05, *** *p* < 0.001 to non-diabetic. # *p* < 0.05, ### *p* < 0.001 to diabetic+vehicle. *n* = 5 to 8 rats per group. Values are mean ± SEM.

Resident retinal microglia, when activated, are a source of pro-inflammatory cytokines that injure the retinal vasculature [24]. To identify microglia, immunolabeling for ionized calcium binding adaptor protein 1 (Iba1) was performed, but this method does not distinguish between microglia and macrophages. In (mRen-2)27 rats, the density of microglia was higher in diabetic+vehicle compared to non-diabetic controls (Figure 4). In diabetic (mRen-2)27 rats, finerenone and perindopril reduced the density of retinal microglia, but only finerenone reduced the expression of the pro-inflammatory mediators, intercellular adhesion molecule-1 (ICAM-1), and interleukin (IL)-1ß in the retina (Figure 4).

### 2.5. OIR: Finerenone Reduced Retinal Neovascularization, VEGF, and Vascular Leakage

**Figure 5:**
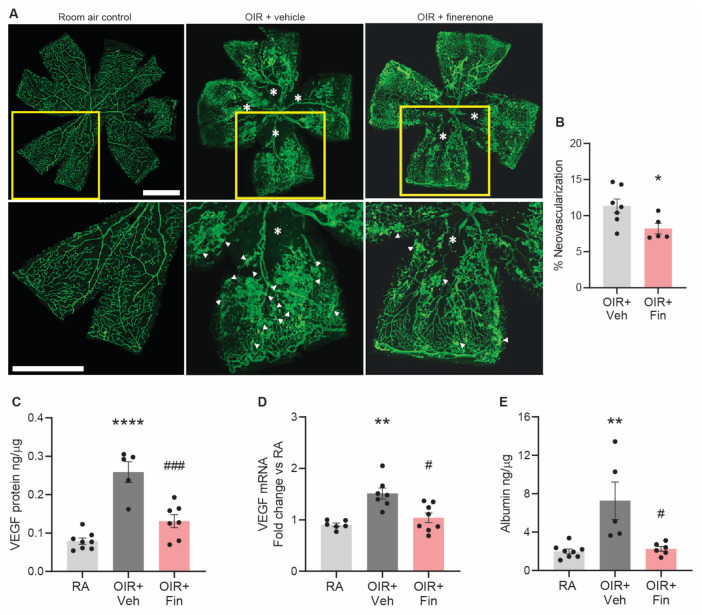
Finerenone reduced retinal neovascularization, VEGF, and vascular leakage in mice with OIR at postnatal day 18. RA, room air control. Veh, vehicle. Fin, finerenone. (**A**) Retinal wholemounts labeled with FITC-isolectin to delineate retinal blood vessels in green. Top panels show whole retina. A quadrant of retina (yellow box) is magnified in the lower panel. Scale bar = 500 µm. Asterisks denote vaso-obliteration. Arrows denote neovascularization. (**B**) Quantitation of retinal neovascularization. * *p* < 0.05 to OIR+vehicle. *n* = 5 to 7 mice per group from 2 to 3 liters per group. (**C**) Retinal VEGF protein levels (ELISA). (**D**) Retinal VEGF mRNA levels. (**E**) Retinal vascular leakage (albumin ELISA). ** *p* < 0.01, **** *p* < 0.0001 to RA. # *p* < 0.05, ### *p* < 0.001 to OIR + vehicle (Kruskal–Wallis test). *n* = 5 to 8 mice per group from 2 to 3 litters of mice per group. Values are mean ± SEM.

We next evaluated the ability of finerenone to influence retinal neovascularization using the murine OIR model. As reported previously [26], mice with OIR had reduced body weight compared to room air controls (Table S1). Finerenone did not influence the body weight of OIR mice (Table S1). As expected, OIR vehicle controls developed retinal neovascularization (Figure 5). Finerenone reduced retinal neovascularization, VEGF protein and mRNA levels, and retinal vascular leakage in OIR mice compared to OIR + vehicle (Figure 5).

### 2.6. OIR: Finerenone Increased Tregs and Reduced Microglial Density

**Figure 6:**
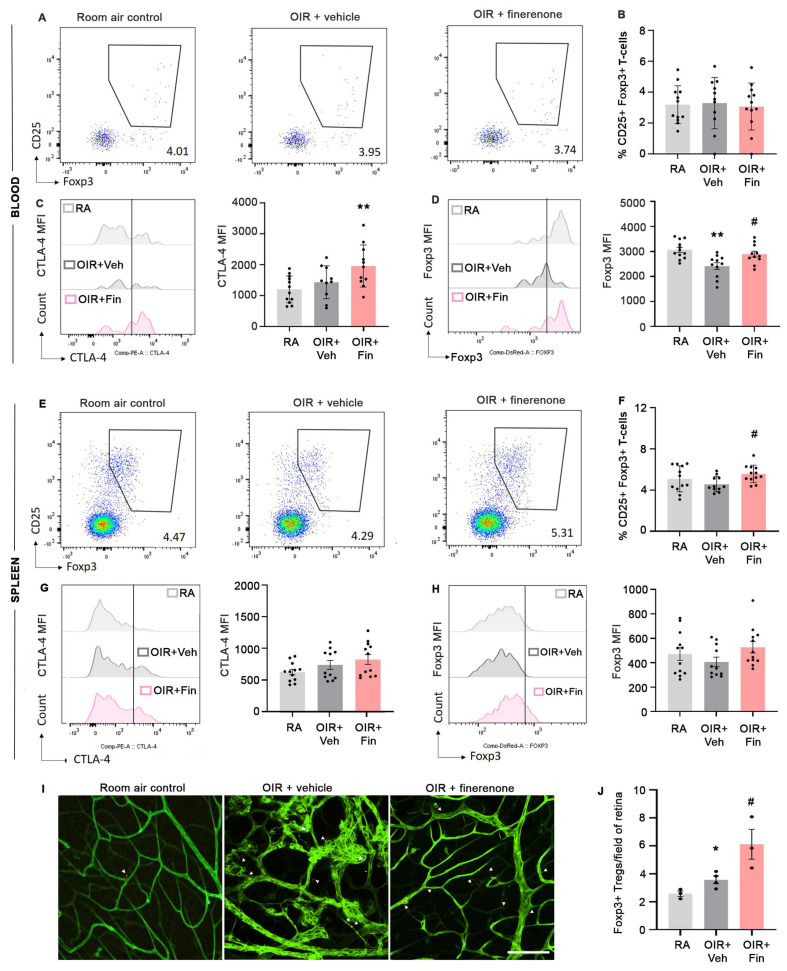
Finerenone increased Foxp3^+^ Tregs in the blood, spleen, and retina of OIR mice at postnatal day 18. RA, room air control. Veh, vehicle. Fin, finerenone. MFI, mean fluorescence intensity. (**A**–**D**) Blood. (**A**,**B**) Dot plots and quantitation of CD25^+^ Foxp3^+^ T-cells. (**C**,**D**) Foxp3 MFI of Tregs. ** *p* < 0.01 to RA. # *p* < 0.05 to OIR+vehicle (one-way ANOVA). (**E**–**H**) Spleen. (**E**,**F**) Dot plots and quantitation of CD25^+^ Foxp3^+^ T-cells. ## *p* < 0.01 to OIR + vehicle (Welch’s and Brown–Forsythe ANOVA). (**G**) CTLA-4 MFI of Foxp3^+^ Tregs. (**H**) Foxp3 MFI of Tregs. *n* = 10 to 12 mice per group from 1 to 2 litters of mice. Values are mean ± SEM. (**I**) Retinal wholemounts from Foxp3^rfp^ mice labeled with FITC-isolectin to delineate retinal blood vessels in green. Scale bar = 200 µm. Foxp3^rfp^-positive cells are shown in red (arrows). (**J**) Quantitation of Foxp3^+^ Tregs in the retina. * *p* < 0.05 to RA. # *p* < 0.05 to OIR + vehicle (one-way ANOVA). *n* = 3 to 4 mice per group from 1 litter per group.

MR antagonists increase the abundance of anti-inflammatory Tregs in cardiorenal disease [40], but whether this occurs in neovascular retinopathy is unknown. As Tregs produced in lymphoid tissues circulate in blood to reach target tissues, we used flow cytometry to determine if finerenone increased the abundance of CD25^+^ Foxp3^+^ Tregs in blood and the spleen. We also evaluated their functionality by measuring the expression of the transcription factor Foxp3 and cytotoxic T-lymphocyte-associated antigen 4 (CTLA-4), a protein involved in the immunosuppressive functions of Foxp3^+^ Tregs [41]. In blood but not the spleen, the abundance of CD25^+^ Foxp3^+^ Tregs was increased with finerenone treatment in OIR mice compared to room air controls and OIR+vehicle (Figure 6). In the spleen but not blood, finerenone increased the expression of Foxp3 and CTLA-4 in CD25^+^ Foxp3^+^ Tregs compared to control groups (Figure 6). We next evaluated if finerenone increased the abundance of Foxp3^+^ Tregs in the retina using Foxp3^rfp^ mice engineered to express Foxp3^+^ cells as red fluorescent protein (rfp). In the retina, the number of Foxp3^+^ cells were slightly increased in OIR+vehicle compared to room air controls, and finerenone treatment further increased Foxp3^+^ cells in the retina of OIR mice (Figure 6). We previously reported that Tregs reduced the density and activity of microglia in the retina of OIR mice [26], and the MR antagonist spironolactone reduced the expression of pro-inflammatory mediators in microglia exposed to hypoxic conditions [42]. Here, we found that finerenone reduced the density of microglia/macrophages in the retina of OIR mice (Figure S2).

## 3. Discussion

The present study is the first to demonstrate the ability of finerenone to reduce components of retinal vascular pathology contributing to vision loss and blindness in DR. Our studies in hypertensive and diabetic rats highlight the efficacy of finerenone delivered by an oral route to reduce breakdown of the BRB and the elevated levels of VEGF involved in the pathogenesis of DR. We report anti-angiogenic actions of finerenone that are relevant to proliferative DR as shown by the reduction in retinal neovascularization and VEGF expression in OIR mice. A mechanism underpinning retinal vascular pathology is the inflammatory environment of the retina [43,44]. The ability of finerenone to attenuate retinal inflammation is evidenced by the reduction in the density of microglia/macrophages and pro-inflammatory mediators and the increased number of anti-inflammatory Foxp3^+^ Tregs in the retina. In diabetic (mRen-2)27 rats, we acknowledge the protective actions of finerenone could be attributed to its anti-hypertensive effects, albeit perindopril reduced systolic blood pressure to a greater extent but did not lower VEGF, ICAM-1, and IL-1ß levels compared to diabetic controls. A limitation of our study is that we did not discern whether the MR expressed in cell types involved in maintaining the BRB such as vascular endothelial cells, pericytes, and macroglial Müller cells, and modulating inflammation such as microglia and Tregs [45,46], contributed to the retinoprotection afforded by finerenone. Nevertheless, our findings, in the context of the positive outcomes of the FIDELIO-DKD and FIGARO-DKD trials involving the oral administration of finerenone [19,20], indicate the potential of finerenone as a treatment for DR. In fact, two sub-studies of identical observational design (ReFineDR and DeFineDR) retrospectively collected data from patients with DR in FIDELIO-DKD/FIGARO-DKD at selected centres [47]. After 2 years of treatment, there was a trend for a lower proportion of patients that had experienced a vision-threatening complication with finerenone compared to placebo and fewer patients required ocular interventions with finerenone. These observed trends for benefit of finerenone on the progression of DR were independent of glycated haemoglobin (HbA1c) levels [47].

The intraocular administration of anti-VEGF agents has revolutionized the treatment of DR by protecting the BRB and attenuating macula oedema and retinal neovascularization [48]. However, there are limitations to this approach including the requirement for frequent intravitreal injections, the lack of adequate response by some patients, and treatment not preventing the progression of DR [49]. One of the earliest pathological events in DR is the response of Müller cells to hyperglycaemia resulting in a reactive gliosis [37,50] that is followed by their increased production of VEGF, which promotes vascular permeability [38]. In diabetic (mRen-2)27 rats, finerenone reduced Müller cell gliosis and VEGF expression in Müller cells and ganglion cells [39], and vascular leakage, suggesting a protective effect of finerenone on the BRB. The finding that retinal VEGF expression and vascular leakage were not increased in diabetic (mRen-2 rats)27 rats compared to non-diabetic controls is likely due to the effect of hypertension on the retina [51]. Finerenone treatment has not been previously evaluated in DR, and in this context MR antagonism has not been extensively studied. Our findings are consistent with a prior study in Goto-Kakizaki rats with type-2 diabetes demonstrating that the intraocular administration of spironolactone reduced retinal vascular leakage and oedema, although retinal VEGF levels were not reduced [29]. There is growing evidence that MR antagonists have benefits in other ocular diseases, attenuating vascular leakage and oedema in central serous chorioretinopathy (CSCR), a condition affecting the outer retina and BRB [52]. Furthermore, aldosterone induced vasodilatation and leakage of choroidal vessels in rats was reduced with MR antagonism, and oral eplerenone reduced subretinal fluid accumulation and resolved retinal detachment in patients with CSCR [53]. Overall, our findings suggest that finerenone has the capacity to protect vision-threatening damage to the BRB in diabetes.

Neovascularization is a central feature of retinopathies such as DR [23], retinopathy of prematurity [54], and neovascular age-related macular degeneration (AMD) [55] that can lead to oedema, haemorrhage, and vision loss. Although not fully explored, MR antagonism has anti-angiogenic effects in retinal vascular diseases. We reported that spironolactone reduced tubulogenesis of cultured retinal endothelial cells and neovascularization in OIR [28]. Furthermore, spironolactone and eplerenone reduced choroidal neovascularization in a rat model of AMD [56], and oral spironolactone reduced signs of choroidal neovascularization in patients with AMD [56]. Our findings that finerenone attenuated neovascularization in mice with OIR and the elevated levels of VEGF mRNA and protein within the retina are consistent with the postulate that finerenone has anti-angiogenic actions relevant to the pathogenesis of DR.

Both DR and OIR are inflammatory conditions [43,44]. In the early stages of disease, leukocytes adhere to the retinal vasculature due to the increased expression of adhesion molecules such as ICAM-1 [57]. The outcomes are areas of tissue non-perfusion and damage to the BRB. In addition, retinal microglia in response to hyperglycemia and ischemia can proliferate and release cytokines such as IL-1ß [58] which injure the vasculature [59]. In the current study, finerenone reduced the up-regulation of ICAM-1 and IL-1ß in the retina of diabetic rats, and microglia/macrophage density in both DR and OIR. These findings are consistent with the documented pro-inflammatory actions of aldosterone in cardiorenal tissues that are reduced by treatment with MR antagonists [9,10,60]. Aldosterone has similar actions in the retina, increasing leukocyte adhesion in OIR that is attenuated by spironolactone [28]. Furthermore, aldosterone increased the infiltration of phagocytes in a model of retinal vein occlusion [61], and spironolactone reduced the hypoxia-induced increase in VEGF, CCL5, and interferon δ in primary cultures of retinal microglia [42].

We reported that strategies to increase the abundance of Foxp3^+^ Tregs resulted in their trafficking from lymphoid organs and blood into the retina, where they reduced the pro-inflammatory phenotype of microglia and repaired the vasculature in OIR [26]. There is a wealth of evidence that T-cells express the MR [46] and MR activation promotes the differentiation of CD4^+^ T-cells to pro-inflammatory Th1 and Th17 cells while decreasing the number of anti-inflammatory Foxp3^+^ Tregs [62]. MR antagonists can influence the abundance of Tregs and have been explored in a model of deoxycorticosterone acetate (DOCA) salt-induced organ damage, where spironolactone attenuated cardiac and renal damage by reducing the activation of Th17 cells and preventing the downregulation of Tregs [40]. Recently, finerenone was shown to prevent cardiorenal damage in a hypertensive DOCA-salt mouse model and reduce the accumulation of IL-17-producing RAR-related orphan receptor gamma (RORδ)-positive γδ T-cells in kidney [63]. In the present study in OIR mice, finerenone increased splenic CD25^+^ Foxp3^+^ T-cells and circulating Foxp3^+^ Tregs expressing their surface marker CTLA-4 responsible for maintaining T-cell homeostasis. This was accompanied by an increase in Foxp3^+^ Tregs in the retina, suggesting Tregs had migrated to the retina and contributed to protecting the vasculature in OIR. The reduction in the density of microglia in OIR with finerenone treatment suggests that Foxp3^+^ Tregs might have directly interacted with microglia via cell surface co-stimulatory molecules to reduce inflammation as previously described [26], although this is yet to be confirmed.

In conclusion, we report that finerenone has beneficial effects in the retina of rodents with diabetes and ischemic retinopathy, reducing the hallmark features of vision-threatening vascular injury that include breakdown of the BRB and neovascularization, as well as a reduction in retinal inflammation. These findings suggest that finerenone might be a potential new oral treatment for patients with DR.

## 4. Materials and Methods

### 4.1. Animals

All experiments were approved by the University of Melbourne Animal Ethics Committee (Applications 10280 and 10485, School of Biomedical Sciences) and performed according to the Australian Code for the Care and Use of Animals for Scientific Purposes. All animals were housed at 22°C in a 12-hour light/dark cycle and had free access to water and food. Investigators were blinded to the experimental groups. In diabetic studies, female transgenic (mRen-2)27 rats at 6 to 8 weeks of age (150–200 g) were randomly allocated to experimental groups. Females were studied as males develop advanced cardiovascular disease [64]. Rats were administered a single tail vein injection of streptozotocin (55 mg/kg, Sigma-Aldrich, CA, USA) diluted in vehicle (0.1 M citrate buffer, pH 4.5) to become diabetic or vehicle to become non-diabetic. Diabetic rats had blood glucose levels measured in the morning on three separate days each week by obtaining a drop of blood from the tail which was applied to the Accu-check Advantage II Blood Glucose Monitor (Roche Diagnostics, USA). The rats had free access to food and water prior to the measurement of blood glucose. Insulin was provided as required (1 to 4 units s.c., Humulin NPH, Eli Lily and Co., Indianapolis, IN, USA). Diabetic rats were excluded from the study if their blood glucose levels were lower than 15 mmol/L. Diabetic rats were randomized to receive, for 12 weeks, finerenone (10 mg/kg/day) or the vehicle for finerenone (40% kolliphor HS 15, Sigma-Aldrich, St Louis, USA, 10% ethanol, 50% water) each day by oral gavage based on previous studies [65]. Comparisons were made to diabetic rats administered the angiotensin-converting enzyme inhibitor perindopril in drinking water for 12 weeks (10 mg/kg/day in sterile water). Body weight was measured weekly, and systolic blood pressure by tail cuff plethysmography (Coda, Kent Scientific, Connecticut, USA) at the end of the study.

OIR was used to investigate the proliferative stage of DR and also retinopathy of prematurity in children [54], and was performed according to our previous studies [26]. Male and female C57BL/6J mice at postnatal day 7 and their nursing mothers were placed in a hyperoxia chamber (75% O_2_) cycled with 2 hours of room air (22% O_2_) each day for 5 days. On postnatal day 12, mice were placed in room air until postnatal day 18 to induce retinal neovascularization. Only mothers with 5 to 7 pups were studied to ensure optimal body weight gain. Mice housed entirely in room air served as room air controls. Tissues and blood from male and female mouse pups were pooled as OIR develops to the same extent in both sexes [66]. As oral administration of finerenone is not possible in neonatal mouse pups, finerenone was administered by i.p. injection. OIR mice were administered finerenone (5 mg/kg/day) or vehicle by i.p. injection each day between postnatal days 7 and 18. To study Foxp3^+^ Tregs, mice engineered to express Foxp3 as a red fluorescent protein (rfp) (Foxp3^rfp^ mice) and bred on a C57BL/6J background were utilized [26]. At the end of the diabetes and OIR studies, animals were humanely euthanized with sodium pentobarbitone (170 mg/mL, Virbac, NSW, Australia) and the blood, spleen, and retina were collected.

### 4.2. Gliosis

Briefly, three-micrometer paraffin sections were incubated overnight at 4 °C with a rabbit polyclonal anti-GFAP antibody (1:500, Z0334, DakoCytomation, Glostrup, Denmark). The sections were then washed with 0.1 M phosphate buffered saline (PBS, pH 7.4) and incubated for 1 h with Alexa Flour 488-conjugated goat anti-rabbit IgG (1:200, A-11008, Life Technologies, Victoria, Australia). The sections were washed with PBS, counterstained with 4′,6-diamidino-2-phenylindole (DAPI, 0.5 µg/mL, D9542, Sigma-Aldrich, St Louis, Missouri, USA), and coverslipped with Dako fluorescent mounting medium (S3023, DakoCytomation). For quantitation, six sections at least 60 μm apart were randomly selected from one eye from each animal. In each section, 6 non-overlapping fields spanning the entire retina were captured at x400 magnification using a Nikon DS-Ri2 camera (Nikon Instruments Inc. NY, USA). ImageJ software was used to set a threshold for immunolabeling which was applied to all fields. Data are presented as the percentage of GFAP immunolabeling per field of retina. Investigators were blinded to the experimental groups. Four to six rats per group were evaluated.

### 4.3. VEGF Immunolabeling

Immunohistochemistry for VEGF was performed by incubating three-micrometer paraffin sections with normal donkey serum for 1 h (D9663, Sigma-Aldrich), and then goat anti-rat VEGF (1:500, 564-RV, R&D Systems, Minneapolis, Minnesota, USA) overnight at 4 °C. A negative control without the primary antibody and an isotype IgG control were included. Sections were washed with PBS, incubated for 30 min with biotin-conjugated donkey anti-goat IgG (1:500, 705-065-147, Jackson ImmunoResearch, Pennsylvania, USA), washed with PBS, and then incubated with the Vectastain ABC standard kit (Vector Laboratories, Pennsylvania, USA) for 30 to 45 min and liquid DAB+substrate chromagen system (Dakocytomation) for 15 s. The sections were counterstained with Mayer’s Haematoxylin and coverslipped. Quantitation was performed as described for GFAP. Data are presented as the percentage of VEGF immunolabeling per field of retina. Four to five rats per group were evaluated.

### 4.4. Microglia/Macrophages

The method described for VEGF immunolabeling was followed, except the primary antibody was Iba1 as previously described [67]. Three-micrometer paraffin sections were incubated overnight at 4°C with 10% normal goat serum (5425S, Cell Signaling Technology, Massachusetts, USA) followed by overnight incubation at 4°C with a rabbit anti-Iba1 antibody (1:1000, 019-19741, Wako, Tokyo, Japan). Sections were washed with PBS, incubated for 1 h with biotin-conjugated goat anti-rabbit IgG (1:200, E0432, DakoCytomation), washed with PBS, and then incubated with the Vectastain ABC standard kit (Vector Laboratories) for 30 min and liquid DAB+substrate chromagen system (Dakocytomation) for 15 s. Sections were counterstained with Mayer’s Haematoxylin. Quantitation was performed as described for GFAP. Data are presented as the percentage of Iba1 immunolabeling per field of retina. Four rats and four to seven mice per group were evaluated.

### 4.5. Vascular Leakage

Albumin levels were quantified in the retina and vitreous fluid using a rat albumin ELISA kit (E-25AL, Immunology Consultants Laboratory, Portland, Oregon, USA) or a mouse albumin ELISA kit (E-90AL, Immunology Consultants Laboratory) according to the manufacturer’s instructions and expressed as total protein concentration [26,50]. Seven to eight rats, and six to eight mice, were analyzed in duplicate per group.

### 4.6. Retinal VEGF Protein

Retinas were homogenized with a bullet blender 24 Gold (Next Advance, NY, USA) in T-PER buffer (78510, ThermoFisher Scientific, Scoresby, Victoria, Australia) containing 5 mM EDTA (AM9260G, ThermoFisher Scientific, Scoresby, Victoria, Australia) and protease/phosphatase inhibitor cocktail (1:100, Sigma-Aldrich, St. Louis, MO, USA). Total protein concentration was quantified via colorimetric assay (5000207 & 5000205 Bio-Rad Laboratories, CA, USA). VEGF was measured in retinal lysates using a commercially available ELISA kit (DY493, R&D Systems). Five to eight mice were analyzed in duplicate per group.

### 4.7. Quantitative Real-time PCR

Total RNA from a single retina was isolated using an RNeasy mini kit (Qiagen, Doncaster, VIC, Australia). Then, DNase treatment using TURBO DNA-free TM kit (Ambion, CA, USA) and reverse transcription (First Strand cDNA synthesis kit, Roche, Switzerland) were performed on 500 ng of RNA. 18s rRNA was used as an endogenous control to normalize mRNA expression. To quantify the mRNA expression, relative fold difference in expression was quantitated using the comparative 2-∆∆Ct method. The primer sequences for mouse 18s rRNA are forward primer: 5′-TCGAGGCCCTGTAATTGGAA-3′ and the reverse primer: 5′24 CCCTCCAATGGATCCTCGTT-3′. The primer sequences for VEGFA are forward primer: 5′-AGCAGAAGTCCCATGAAGTGATC-3′ and the reverse primer: 5′TCAATCGGACGGCAGTA-3′. The primer sequence for rat intercellular adhesion molecule-1 (ICAM-1) are forward primer: 5′-’TGAAGATGACGAGACGCAGAGT-3′ and the reverse primer: 5′-CCCAATGTGGCCAAATCC-3′ and rat IL-1ß are: forward: 5′-TCTCCAGTCAGGCTTCCTGT-3′ and the reverse primer: 5′AGGTCATTCTCCTCACTGTCGAAA-3′. Data are presented as the relative fold difference in expression calculated via the 2-ΔΔCT calculation. Five to eight mice/rats per group were evaluated.

### 4.8. Retinal Neovascularization

Retinal neovascularization was measured according to our published methods [26]. Eyes from mice with OIR at postnatal day 18 were enucleated and fixed in 4% paraformaldehyde in PBS for 30 min at room temperature. Retinal flat-mounts were stained with FITC-conjugated isolectin GS-IB4 (Sigma-Aldrich) and imaged using a Zeiss Axio (Zeiss, Jena, Germany) microscope attached to a camera (AxioCam MRc, Carl Zeiss, Gottingen, Germany). Entire retinal montages were obtained using the tiling tool in the AxioObverser Software (v5.3, Zeiss, Jena, Germany). ImageJ and the threshold tool were used to quantitate neovascularization. Between 5 and 7 OIR mice from 2 to 3 liters per group were investigated.

### 4.9. Flow Cytometry for Tregs in the Blood and Spleen

As strategies to increase Foxp3^+^ Tregs can be associated with their increased abundance in the blood and spleen, both were studied in OIR mice and room air controls as previously described [26]. Briefly, spleens were mechanically disrupted and sieved through a 40 mm strainer to generate single-cell suspensions. For blood and spleen cells, 1^x^ RBC lysis buffer (#00-4333-57, eBioscience, ThermoFisher Scientific) was used to remove red blood cells. One million cells were incubated with antibody cocktails CD3 FITC (#100306, Biolegend, Maryland, USA), CD4 APC (#17-0042-81, eBioscience), CD8 BV711 (#100748, Biolegend), and CD25 APC (#561048) for 45 min at 4°C. For intracellular staining of Foxp3^+^ Tregs, cells were treated with the Fixation/Permeabilization solution (#554714, eBioscience) according to the manufacturer’s protocols and stained with the Foxp3 antibody (Foxp3-PE-eF610, #61-5773-82, eBioscience) and CTLA-4 antibody (CTLA-4-PE, #61-5773-82, eBioscience) for 45 min at 4°C. Six to twelve mice per group were evaluated. The gating strategy for flow cytometry is found in Figure S2.

### 4.10. Quantitation of Tregs in the Retina

Foxp3^+^ Tregs in the retina were evaluated as previously described [26]. Eyes from litters of Foxp3^rfp^ mice at postnatal day 18 were enucleated and fixed in 4% paraformaldehyde in PBS for 30 min. Eyes were immediately flat-mounted, and stained with FITC-conjugated isolectin GS-IB4 (Sigma-Aldrich) overnight at 4°C. Foxp3^+^ Tregs were imaged with a confocal microscope (Zeiss LSM880) at 100× magnification in the superficial retinal layers (inner limiting membrane and ganglion cell layers). Twelve fields per retina were imaged, and the number of Foxp3^+^ Tregs was counted and expressed as the number of cells per field. Investigators were blinded to the experimental groups. Three to four mice per group were evaluated.

### 4.11. Statistics

All data were assessed using GraphPad Prism software (V9.3.1, San Diego, CA, USA) and were subjected to a normality test using the Shapiro–Wilk test and then cross-validated with Kolmogorov–Smirov, D’Agostinos, and Pearson omnibus tests to avoid false positives. Data with normal distribution were assessed using one-way ANOVA followed by Tukey’s test. The nonparametric Kruskal–Wallis test followed by Dunn’s post-test was used for data that did not pass the normality test. For comparisons between two groups, a *t*-test was used for data that passed the normality test and Mann–Whitney U test for data that did not pass. Values are presented as mean ± SEM.

## Data Availability

The data presented in this study are available on request from the corresponding author.

## Supplementary Materials

The following supporting information can be downloaded at: https://www.mdpi.com/article/10.3390/ijms24032334/s1.
