# Supplementary material for: Finerenone, a Non-Steroidal Mineralocorticoid Receptor Antagonist, Reduces Vascular Injury and Increases Regulatory T-Cells: Studies in Rodents with Diabetic and Neovascular Retinopathy
Source: Int J Mol Sci. 2023 Jan 25;24(3):2334. doi: 10.3390/ijms24032334 (PMC9917037; doi:10.3390/ijms24032334)
Supplement: Supplementary file 1 [file ijms-24-02334-s001.zip › ijms-2095819-supplementary.pdf]

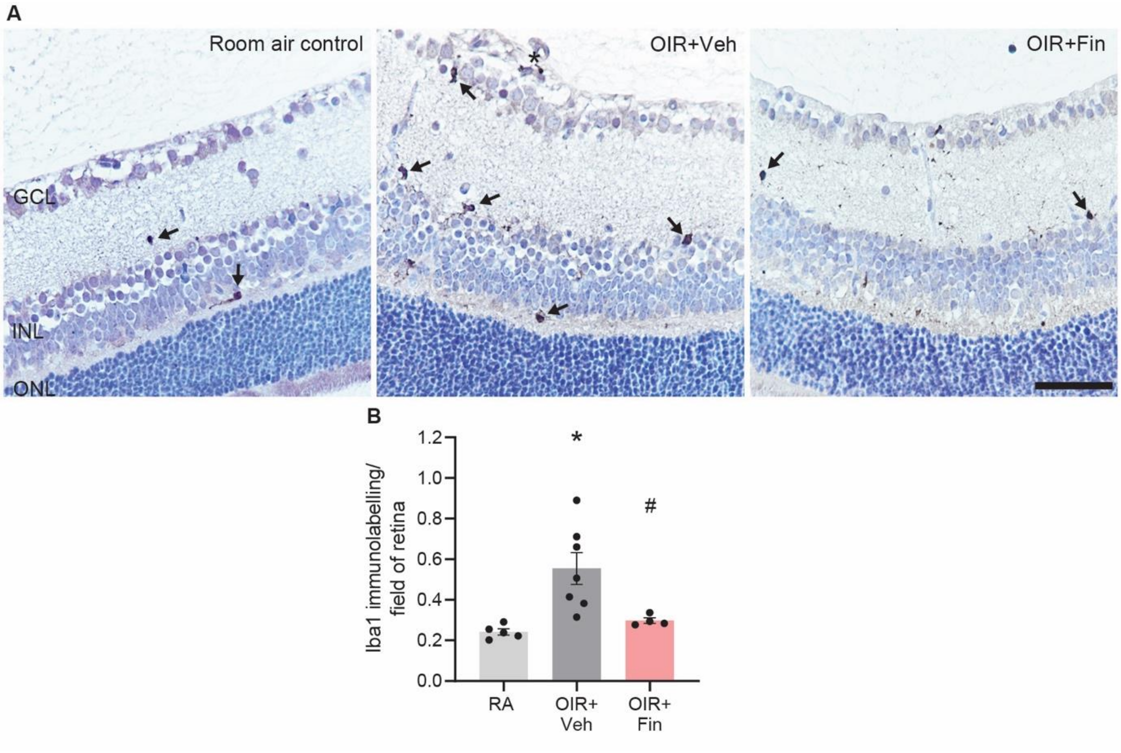

Figure S1. Finerenone reduced microglia/macrophage density in retina of mice with OIR at postnatal day 18. RA, room air control. Veh, vehicle. Fin, finerenone. GCL, ganglion cell layer. INL, inner nuclear layer. ONL, outer nuclear layer. A. Three µm paraffin sections of retina immunolabeled with Iba1 to detect microglia/macrophages. Counterstain, hematoxylin. Scale bar = 50 µm. Iba1 labelled microglia/macrophages are denoted by arrows. Neovascular tufts are denoted by an asterisk. B. Iba1 quantitation. *$p < 0.05$ vs room air control. #$p < 0.05$ to OIR+vehicle (Welch's and Brown-Forsythe ANOVA). $n = 4$ to 7 mice per group. Values are mean ± SEM.

**Group**	**n**	**Body Weight**
Room air control	19	8.2 ± 0.89
OIR + vehicle	20	7.3 ± 0.59**
OIR + finerenone	14	7.4 ± 0.49**

Table S1. Body weight of OIR mice at postnatal day 18. **$p < 0.01$ to room air controls (Welch's and Brown-Forsythe ANOVA). $n = 14$ to 20 Foxp3^{rfp} mice from 3 litters per group. Values are mean ±SEM.

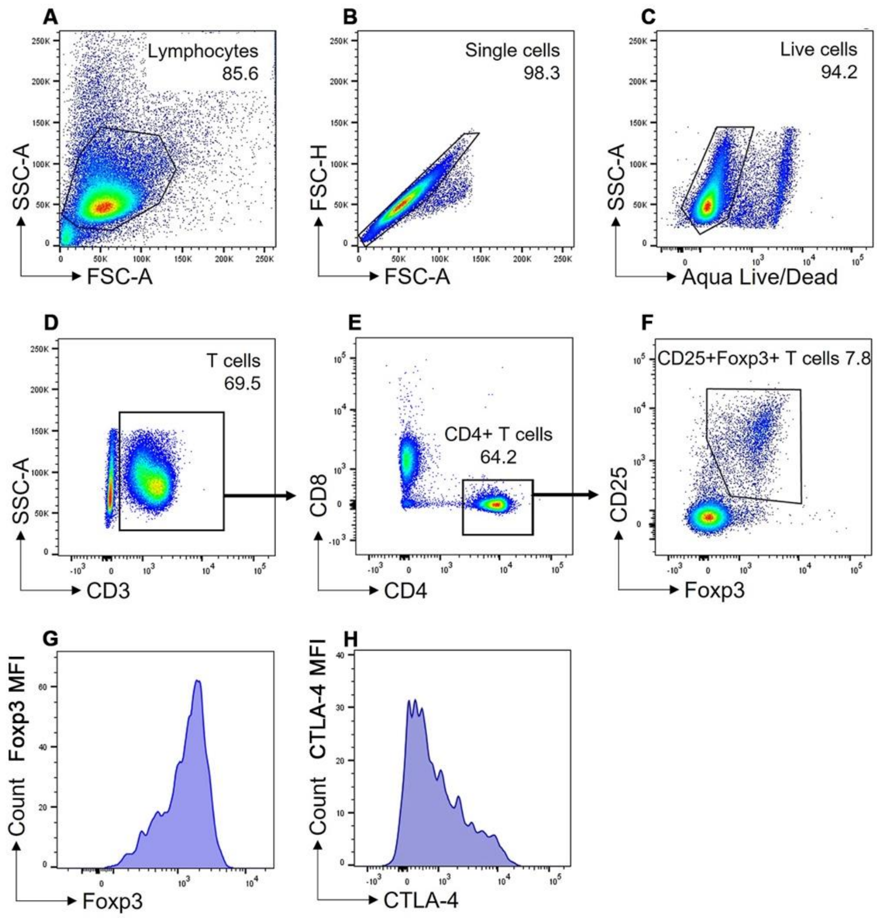

Figure S2. Gating strategy to identify Foxp3+ Tregs with flow cytometry in the spleen of mice. SSC, side scatter. FSC, forward scatter. MFI, mean fluorescence intensity. (**A**). Lymphocytes. (**B and C**). Duplets and dead cells were eliminated from the lymphocyte population. (**D and E**). CD3+ T cells and CD4+ T cells were selected. (**F**). From this population CD25+ Foxp3+ T cells were selected to analyze the presence of Foxp3 and CTLA-4. (**G**). Foxp3 MFI of Tregs. (**H**). CTLA-4 MFI of Foxp3+ Tregs.
